# Collective efficacy measures for women and girls in low- and middle-income countries: a systematic review

**DOI:** 10.1186/s12905-022-01688-z

**Published:** 2022-04-25

**Authors:** Nabamallika Dehingia, Anvita Dixit, Karen Heskett, Anita Raj

**Affiliations:** 1 Center On Gender Equity and Health, School of Medicine, University of California San Diego, 9500 Gilman Drive #0507, La Jolla, CA 92093-0507 USA; 2 Joint Doctoral Program in Global Health, San Diego State University and University of California San Diego, San Diego, CA USA; 3 Biomedical Library, University of California San Diego, San Diego, CA USA

**Keywords:** Collective efficacy, Measurement, LMIC

## Abstract

**Background:**

Prior research has shown collective efficacy to be a key determinant of women’s well-being. However, much of the work around measuring this construct has been done in high-income geographies, with very little representation from low- and middle-income countries (LMIC). To fill this gap, and guide future research in low resource settings, we aim to summarize best evidence measures of collective efficacy for women and girls from LMICs.

**Methods:**

Following PRISMA guidelines, we systematically searched five databases for English language peer-reviewed literature on measures of collective efficacy, published between 1 January 2009 and 25 August 2020. In addition, we sought expert input for relevant papers in this area. Research staff screened titles, abstracts, and full-text articles in a double-blind review. Inclusion criteria were: (i) original quantitative analysis, and (ii) sample limited to women/girls only (≥ 100), residing in LMICs.

**Results:**

We identified 786 unique articles, 14 of which met inclusion criteria. Eligible studies captured a diversity of population groups, including pregnant women, recent mothers, adolescent girls, and female sex workers, from across national settings. Two broad constructs of collective efficacy were captured by the measures: (i) group dynamics, and (ii) collective action. All 14 studies included items on group dynamics in their measures, whereas seven studies included items on collective action. Four studies validated new measures of collective efficacy, and seven provided evidence supporting the relationship between collective efficacy and outcomes related to women’s well-being. Overall, measures demonstrated good reliability and validity when tested, and those testing for associations or effects found a positive relationship of collective efficacy with women’s health behaviors.

**Conclusion:**

The past decade has resulted in a number of new collective efficacy measures demonstrating good validity in terms of their associations with key health outcomes among women and girls from across LMIC settings, but there remains no standard measure in the field. Those that exist focus on group dynamics, but less often on collective action. A standard measure of collective efficacy inclusive of group dynamics and collective action can support better understanding of the value of women’s collectives across national settings and populations.

**Supplementary Information:**

The online version contains supplementary material available at 10.1186/s12905-022-01688-z.

## Background

Women’s collectives have been shown to be an important driver in improving women’s sexual, reproductive, and maternal health, child health, and women’s safety from gender- based violence [1–4]. These studies built upon research documenting the value of women’s collectives for their financial security and livelihood [5–7], as well as political participation [8]. Systematic reviews of the literature on effectiveness of collectives highlight that the mechanism though which they achieve these outcomes could be via improved collective efficacy [9–11]. Collective efficacy is also regarded as an essential component of the gender empowerment process as it creates power and agency to develop and maintain group level change; it has a direct bearing on population health and socio-economic indicators, especially for women and girls [12]. Unfortunately, lack of a standard measure of collective efficacy for use in low- and middle-income countries (LMIC), and specifically for women and girls, has likely hindered our understanding of collectives.

Measurement of collective efficacy requires clarity of the concept. In the field of psychology, Bandura defines collective efficacy as, "a group's shared belief in its conjoint capability to organize and execute the courses of action required to produce given levels of attainment" [13]. Collective efficacy relies on both group capacities, and the processes toward achievement of shared goals [14, 15]. A significant amount of research on collective efficacy has focused on its neighborhood-level prevalence, in the context of crime prevention. Sampson et al. [16] has defined it as “social cohesion among neighbors combined with their willingness to intervene on behalf of the common good”. Thus, while social networks and social capital are the foundation of collective efficacy, a notion central to this construct is the desire to intervene as a group, and a capacity for informal control. Prior research posits that collective efficacy is best captured by the two distinct yet overlapping constructs, *social group dynamics* and *desire to take action*, which are in turn influenced by elements of social capital, empowerment, and civic engagement [17]. Group dynamic characteristics can include social support, solidarity, connection, group engagement, dialogue, trust, decision-making and deliberation, identification with the group, and shared goals. These factors are directly related to the desire to take action or collective action; at a community or group level, the desire and capacity to intervene for the common good will depend on the extent of solidarity, engagement, and mutual trust within the group members [18]. Socially cohesive communities encourage the practice or implementation of collective action. In contrast, a member is unlikely to be willing to intervene or participate in collective action in a context where people mistrust or fear each other, and experience a lack of solidarity. In the current study, we review articles that include measures related to either one, or both sub-constructs of collective efficacy- group dynamics, and collective action.

Prior research has noted collective efficacy to be situated rather than global; efficacy is present relative to a specific task, or a type of task [16, 19]. As such, measures should, and will vary based on the context in which collective efficacy is studied. Majority research has viewed collective efficacy as existing relative to neighborhood-level crime, and civic problems. Collective efficacy in the context of women's issues, or women's rights, is a relatively lesser focused field of work. Although, understanding the dimensions of group dynamics and collective action are particularly important for socially disadvantaged and politically disenfranchised groups, including women, given the value of collectives in providing “safety in numbers”. The existing measures of collective efficacy specifically for women groups, have largely come from high-income countries [16, 20–22]. Analysis of collective efficacy has been more limited in LMICs due to lack of established measures, and lack of rigorous measure development from within these countries [9]. Given the socially and culturally specific nature of collective efficacy as a phenomenon, research on using and developing context specific measures from LMICs is needed, particularly for purposes of monitoring and evaluation [23].

To support the use of best evidence measures, as well as to inform development of new measures for this important and growing area of work, we conducted a systemic review of the literature, and utilized expert input to identify measures of collective efficacy for women and girls in LMICs. We assessed the nature and psychometrics of identified measures, and the population of focus. In order to provide a comprehensive understanding of the context in which the different measures have been implemented, where available, we also examined the application of collective efficacy measures to outcomes for women's well-being.

## Methods

We conducted a systematic review of the literature to assess quantitative measures of collective efficacy, following the PRISMA Statement checklist [24]. The predefined protocol and full search strategy are available online, in our PRISMA registration and protocol [25].

### Literature search

Five electronic bibliographic databases [PubMed (pubmed.gov), Embase (embase.com), PsycINFO (ProQuest), Sociological Abstracts (ProQuest), and Family & Society Studies Worldwide (EBSCO)] were searched for peer-reviewed literature related to measures of collective efficacy, published from January 2009 to August 25, 2020. Our search strategy was developed by a library subject specialist, and combined terms related to community, social capacity and action, LMICs, tests or measurement tools, and psychometrics, or validity and reliability. Search terms were developed to characterize collective efficacy based on review of theoretical concepts of collective efficacy which posit that efficacy is captured by measures of capacity (thinking you can do it and being able to do it) and action [14, 16, 26, 27]. Our search strategy is provided in Additional file 1. To identify additional studies, we gathered input from experts working on collective efficacy and gender empowerment, and are a part of the larger Evidence based Measures of Empowerment for Research on Gender Equality (EMERGE) project at the Centre in Gender Equity and Health at University of California San Diego. EMERGE maintains a large repository of existing measures related to women and girls’ health, with the broader goal of identification and evaluation of quantitative measures of gender equality and empowerment [28].

### Inclusion and exclusion criteria

Studies were included if they showed evidence of any original quantitative data analysis (including but not limited to psychometric properties i.e. reliability or validity) on collective efficacy, and the sample size was women/female only, with at least 100 participants in an LMIC setting. We excluded studies which had no quantitative data analysis (e.g. only provides qualitative data, case study, reviews of literature etc.), had sample size less than 100 participants, or had population groups that included men, or men and women together, were not in English language, or were conducted in non-LMIC only, or were clearly off topic.

### Data extraction and quality assessment

Studies were reviewed by trained research assistants who used Covidence to review their eligibility, following a double-blind review process [29]. Any disagreements were resolved by the senior author of the study. The technology used on our project included the Yale MeSH analyzer as well as the duplicate detection capability of Covidence. We also used EndNote for pulling full-text combined with a tool called FolderMerge to allow for a bulk upload of PDFs to Covidence. Due to the non-clinical or experimental nature of this review, an established quality assessment guideline was not practicable. However, the Strengthening the Reporting of Observational Studies in Epidemiology (STROBE) Statement, guidelines for reporting observational studies, were adapted to ensure key aspects of the studies were assessed during data extraction to indicate quality of the studies [30]. We conducted a qualitative synthesis of the findings and extracted the following information from each study: year of study, research question, study design, study sample including study settings, measures in the study that are about, or related to collective efficacy, psychometrics for the relevant measures, study results, and key concepts of collective efficacy. Based on above described theoretical understanding, we focused on the following main constructs and sub-constructs of collective efficacy: *Construct 1. Group dynamics* (including group engagement, support, solidarity, connection, dialogue, trust, decision-making and deliberation, identification with the group, and shared goals) and *Construct 2. Collective action* (including group organizing, action, leadership, and voice). For psychometrics, we extracted all information provided by the authors regarding reliability and validity of the measures of interest.

## Results

**Fig. 1:**
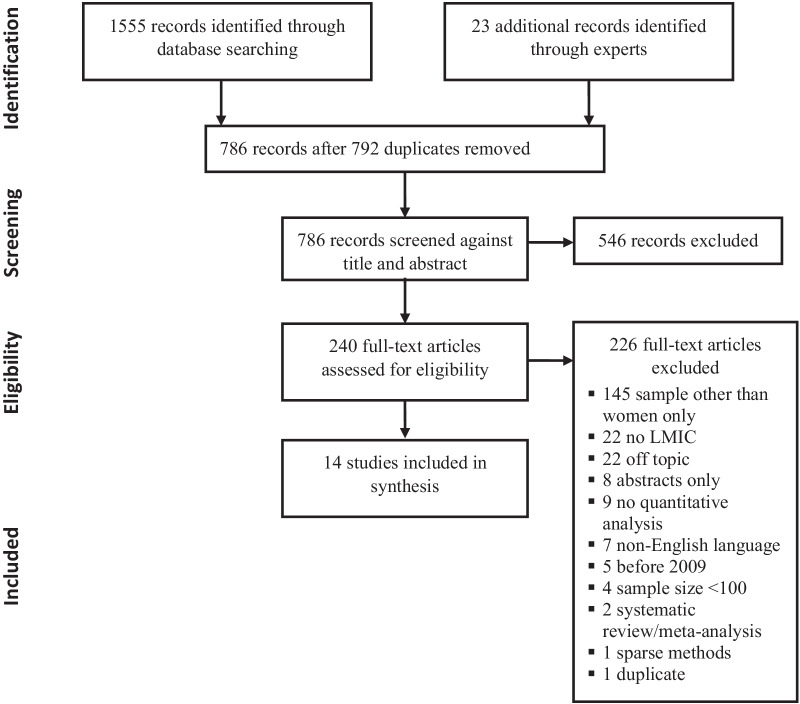
PRISMA flowchart showing study selection process for the systematic review

We identified 1,555 articles via database searching, and 23 articles through expert input. Of these, 786 were unique citations. Two coders independently reviewed these papers, and 14 studies met all inclusion criteria for analysis (Fig. 1).

Population sub-groups: The 14 studies included different population sub-groups, and represented a diverse geography, with countries in South Asia, sub-Saharan Africa, the Middle East, and the Caribbean (Table 1). Three studies included measures for female sex workers (FSW) in India and Dominican Republic [31–33], while two studies used measures for recent mothers in Malawi [34, 35]. Remaining studies covered pregnant women in Sri Lanka [36], self-help group (SHG) members in India [37, 38], female nurses in Iran [39], HIV‐negative adolescent girls in South Africa [40], and general adult female population in Ghana, Uganda and Malawi [41], Iran [42], China [43], and India [44]. All except one [40] focused on adult women, with the average age ranging from 15 to 40 years across studies.

**Table 1:** Eligible studies from systematic review of collective efficacy measures in low- and middle- income countries

S.No	References	Research question/aim	Study design	Study sample-means of recruitment, demographics and other characteristics, sample size	Measures of or related to collective efficacy in the paper: include each collective efficacy measure, and for each measure indicate number of items, types of things included in the measure, and response pattern (where available)	Psychometrics of Collective Efficacy Measures	Study results (for studies whose objective was not measures validation)	Key concepts of collective efficacy measured
1	Agampodi et al. [36]	To develop and validate an instrument to measure social capital among pregnant women in low- and middle- income countries—the Social Capital Assessment Tool for Maternal Health (LSCAT-MH)	Cross-sectional Purpose: measures development and validation	Setting: Sri Lanka Purposive sample of 439 pregnant women Age: 85.0% of the women of age 20–35 years Education: 1.3% up to grade 5 or less, 24.2% up to grade 10, and 7.3% had university education	Study developed and validated a 24-item measure of **social capital** for pregnant women which consisted of four factors: • **Neighbourhood networks:** includes *four* items that assess structural bonding, or the informal social networks such as having someone to consult for information/knowledge, meeting friends/relatives in the neighbourhood, connecting with friends on telephone, and having someone to console when stressed • **Domestic and neighbourhood cohesion:** includes *nine* items that assess cognitive bonding such as couple and family arguments, nature of relationship with the neighbourhood with regards to receiving love and support • **Social contribution:** includes *six* items that assess bonding and bridging ties such as taking responsibilities for social activities, and helping others in the community • **Social participation:** includes *three* items that assess structural bridging such as participation in cultural events and showing solidarity in the case of neighbourhood problem Two types of response scales were tested; five-point Likert (Fully agree, agree, neutral, disagree, fully disagree) and five-point adjectival (always, often, sometimes, rarely and never). The adjectival scale performed better as reported by the respondents	**Reliability:** Cronbach's alpha = 0.94 Intraclass correlation (ICC) for test–retest reliability = 0.71 **Validity:** Construct validity established with a significant negative correlation (correlation coefficient, r = − 0.269, *p* = 0.000) with the Edinburgh Postpartum Depression Scale Face validity assessed and found adequate	Not applicable	GROUP DYNAMICS: • Group support • Group solidarity • Positive group dynamics • Social support and connection • Group dialogue • Trust • Identification with the group
2	Firouzbakht et al. [39]	To validate the Persian version of the workplace social capital questionnaire, for a sample of female health care workers in Iran	Cross-sectional Purpose: measures validation	Setting: Iran Multi-stage random sample of 440 female nurses in hospitals and health care centres, with at least one year of work experience Mean age: 35.9 (SD 8.4) years. Education: 77.3% had a bachelor’s degree	Study validated an 8-item measure of **workplace social capital**, which consisted of two factors: • **Group coherence:** includes *five* items that assess whether members of the work unit build on each other's ideas, co-operate, and feel accepted and understood by each other • **Committed management:** includes *three* items that assess the respondents’ relationship with the supervisor in terms of trust and support A five-point Likert scale ranging from 1 to 5 (“totally disagree” to “totally agree”) was used for responses to the items	**Reliability**: Cronbach’s alpha = 0.80 **Validity:** Construct validity established with a two- factor solution that explained 65% of the total variance. Fit statistics were acceptable: GFI = 0.953, CFI = 0.973, RMSEA = 0.090 Content validity established via assessment of the items by experts	Not applicable	GROUP DYNAMICS: • Group support • Group solidarity • Positive group dynamics • Social support and connection • Trust
3	Salehi et al. [42]	To validate the Trust and Control-Self-efficacy scales for a sample of young women living in Iran The original measure was used in the British General Household Survey (GHS)	Cross-sectional Purpose: measures validation	Setting: Iran Cluster convenience sample of 391 women Age range: 18 to 35 years (mean 27.3, SD 4.8 years) Education: 76.4% of women with a university degree, and 49% women single	Study validated a 20-item measure of **Trust**, which consisted of four factors: • **Trust in media**: includes *six* items that assess whether the respondent trusts TV, radio, and local newspapers • **Trust in institutions:** includes *eight* items that assess whether the respondent trusts different institutions including the government, parliament, police, and bank • **Trust in neighbourhood:** includes *four* items that assess whether the respondent trusts their neighbourhood, or believes the neighbourhood is helpful • **General trust:** includes *two* items that assess whether the respondent believes that most people can be trusted A five-point Likert scale ranging from 1 (strongly disagree) to 5 (strongly agree) was used for the first, third, and fourth subscales. The second subscale had Likert response categories ranging from 1 (do not trust at all) to 10 (trust completely). Scores are added for an overall measure	**Reliability**: • Trust in media: Cronbach’s alpha = 0.92 • Trust in institutions: Cronbach’s alpha = 0.93 • Trust in neighbourhood and General trust (combined): Cronbach’s alpha = 0.73 **Validity:** Construct validity established with factor analysis; Trust scale had chi-square value 2.238, CFI 0.967, TLI 0.959, and RMSEA 0.056	Not applicable	GROUP DYNAMICS: • trust
4	Kuhlmann et. al. [35]	To validate measures related to three domains: women’s empowerment, health workers’ empowerment, and negotiated spaces, or engagement between power holders and citizens	Cross-sectional (baseline survey of an RCT evaluation) Purpose: measures validation	Setting: Malawi Cluster sample of 1951 women aged 15–49 who had given birth in the last 12 months Mean age: 25.7 years Education: 30% were illiterate	The first domain, women’s empowerment tested four measures related to collective efficacy: • **Community support in times of crisis:** includes *four* items which asses how sure the woman is about someone in their community supporting them if they are pregnant and bleeding • **Social cohesion:** includes *six* items which assess whether the woman can rely or trust their community members for borrowing money, dealing with a violent situation etc • **Collective efficacy:** includes *four* items that assess women’s confidence in how well community members and health workers could work together for improving health outcomes in the community • **Social participation & collective action:** measured using three separate questions that assess whether the woman has had membership with, or received any help from, an organized group in their community, or joined other people to improve health services The third domain, negotiated spaces, included one relevant measure: • **Mutual responsibility for & support of services:** includes *five* items which ask women who they thought could have the most impact to make changes in five areas related to maternal health including being treated with respect, visits by health worker, and getting funding to improve health services. Options included community members with health workers and government officials, community members alone etc	**Reliability:** • Community support in times of crisis: Cronbach’s alpha = 0.83 • Social cohesion: Cronbach’s alpha = 0.67 • Collective efficacy: Cronbach’s alpha = 0.90 • Social participation & collective action: Cronbach’s alpha = Not applicable (individual questions, not a scale) • Mutual responsibility for & support of services: Cronbach’s alpha = 0.73 **Validity:** All final items had factors loadings > 0.4 in exploratory factor analysis. No other details provided	Not applicable	GROUP DYNAMICS: • Group support • Group solidarity • Positive group dynamics • Social support and connection • Group dialogue • Trust • Group decision-making/deliberation • Shared goals COLLECTIVE ACTION • Group organizing
5	Carrasco et al. [31]	To examine the associations between social cohesion and a) consistent condom use and b) sexually transmitted infections (STIs) among female sex workers (FSW) living with HIV in the Dominican Republic	Cross-sectional (uses data from the follow- up survey of a cohort study) Purpose: association analysis	Setting: Dominican Republic Non-random hybrid sample of 228 FSWs living with HIV, participating in the multilevel intervention *Abriendo Puertas* in 2013 in Santo Domingo Mean age 37 years (range 30–43) Education: 120 (53.91%) had education 0–7th grade, 103 (46.19%) had 8th grade to university	The key independent variable, **social cohesion**, was a 11-item measure that assessed trust, solidarity, and reciprocity among the FSWs Items asked FSWs if they could trust other FSWs with regards to sharing their HIV status, if they could count on other FSW for borrowing money, accompanying to the hospital, finding a place to stay, supporting in use of condoms, and dealing with a violent customer A 5-point Likert-scale was used to capture the response (strongly disagree, disagree, agree, strongly agree, do not know)	**Reliability**: Cronbach’s alpha = 0.81 **Validity:** No statistics for validity are provided in the current study	Study used multivariable logistic regressions to test the hypothesized associations Results show: • Social cohesion significantly associated with consistent condom use (CCU) between FSWs living with HIV and their clients in the last month (adjusted odds ratio (AOR) = 1.65, 95% confidence interval (CI): 1.11–2.45) and STI prevalence among FSWs (AOR: 3.76, 95% CI: 1.159–12.162)	GROUP DYNAMICS: • Group support • Group solidarity • Positive group dynamics • Social support and connection • Trust • Identification with the group
6	Kuhlmann et al. [32]	To examine the associations between strength of community mobilization in a given geographic cluster and a) consistent condom use and b) perceptions of fairness, among FSW	Cross-sectional Purpose: evaluation	Setting: India Convenience sample of 1986 female sex workers from 104 geographic clusters receiving a community mobilization intervention for FSWs Mean age: 29.19 (SD 0.18) Mean education: 3.40 years (SD 0.19). 53% worked in urban areas. 81% had children	The key mediator for the analysis, **collectivisation** (hypothesized as a group of mediating variables for the relationship between community mobilization and outcomes), was assessed via the following variables: • **Collective identity:** includes *a single item* that asked FSWs if they had attended a public event where they could be identified as FSW • **Collective efficacy:** includes *two separate variables*. First variable included *a single item* that asked if FSWs would work together if problem affected the group. The second variable included *four items* that assessed whether the FSWs work well together to achieve their shared goals • **Collective agency:** includes *four items* which asked FSWs if they have stood up for another FSW in need • **Collective action:** includes *seven items* on whether the FSW participant had ever joined the collective to demand entitlements • **Social cohesion:** includes *12 items* on whether the FSW participant had ever shared issues or concerns with other FSWs, or relied on them in any way The key treatment variable, strength of **community mobilization** (i.e., FSW participation in mobilization activities) assessed proportion of target population participating in programme activities, programme implementation, programme management, crisis response, and decision-making, as well as governance processes, leadership, and ownership	**Reliability**: • Collective identity: single item, no Cronbach’s alpha • Collective efficacy: Cronbach's alpha = 0.75 • Collective agency: Cronbach's alpha = 0.76 • Collective action: Cronbach's alpha = 0.80 • Social cohesion: Cronbach's alpha = 0.69 • No reliability statistics provided for community mobilization **Validity:** • No statistics for validity are provided for the measures of collectivisation • The community mobilization measure was validated in development via key informant interviews and organization documents	Study used multi-level structural equation modelling, with propensity score reweighting to simulate a randomized dose–response Results show: •No significant effect of community mobilization on consistent condom use or fair treatment among FSWs •A significant indirect effect of community mobilization on consistent condom use mediated through social cohesion (b = 1.63, SE = 0.75, β = 0.31, *p* < 0.05) A direct effect of strength of community mobilization at the cluster level on collective identity (b = 1.11, SE = 0.45, β = 0.40, *p* < 0.05), and social cohesion (b = 0.57, SE = 0.15, β = 0.36, *p* < 0.01)	GROUP DYNAMICS: • Group support • Group solidarity • Positive group dynamics • Social support and connection • Group dialogue • Identification with the group • Group decision-making/deliberation • Shared goals COLLECTIVE ACTION: • Group organizing • Leadership and voice
7	Parimi et. al. [33]	To examine the association between FSWs’ degree of community collectivisation and a) self-efficacy, and b) utilisation of STI services	Cross-sectional Purpose: association analysis	Setting: India Convenience sample of 1986 FSWs from 104 geographic clusters receiving a community mobilization intervention for FSWs Mean age: 29.19 (SD 0.18) Mean education: 3.40 years (SD 0.19) 53% worked in urban areas. 81% had children	The key independent variable, **collectivisation**, was assessed via the following: • **Collective efficacy:** includes *four items* that assessed whether the FSWs work well together to keep each other safe, increase condom use with clients, speak up for their rights, and improve their lives • **Collective agency:** includes *four items* which asked FSWs if they have stood up to police, madam/broker, local goon, and clients or any other sexual partner, for another FSW in need • **Collective action:** includes *six items* on whether the FSW participant had ever joined the collective to demand entitlements such as voters’ card, bank account, and health insurance	**Reliability**: • Collective efficacy: Cronbach's alpha = 0.79 • Collective agency: Cronbach's alpha = 0.76 • Collective action: Cronbach's alpha = 0.76 **Validity:** • No statistics for validity are provided	Study used multivariate logistic regression models to examine the hypothesized relationships Results show: •Collective efficacy increases self-efficacy among FSWs (AOR: 3.8; 95% CI: 2.8—5.1) •Collective agency increases self-efficacy among FSWs (AOR: 2.8, 95% CI: 2.3—3.4) •Collective action increases self-efficacy among FSWs (AOR: 2.5, 95% CI: 1.8—3.5) Overall collectivisation increased the likelihood of seeking STI treatment from government facility	GROUP DYNAMICS: • Group support • Group solidarity • Positive group dynamics • Social support and connection • Group dialogue • Identification with the group • Group decision-making/deliberation • Shared goals COLLECTIVE ACTION • Group organizing • Leadership and voice
8	Story [44]	To examine the association between social capital and the utilization of antenatal care, professional delivery care, and childhood immunization	Cross-sectional Purpose: association analysis	Setting: India Multi-level sample of 10,739 women in 2293 villages or urban neighbourhoods. Mean age: 27.4 years Education: 42% women never attended school 5% belonged to General caste and 79% Hindus	The key independent variable of the analysis, social capital, included six factors: • **Intergroup bridging ties:** includes *five* items and examines the respondents' membership in development groups, including women's groups; youth clubs, sports groups, reading rooms; trade unions, business or professional groups; self-help groups; and credit or savings groups • **Intragroup bonding ties:** includes *two* items and examines the respondents' membership in any religious, caste, or festival organization • **Political participation:** includes *two* items that measured whether the respondent or anyone in the household attended a public meeting and whether anyone in the household is an official of the local committee • **Social networks:** includes *three* items and examines if the respondent knows or is related to any person of importance like doctors, teachers or government officials • **Social cohesion:** includes *two* items and examines if there is any conflict in the respondents' community • **Collective efficacy:** includes *a single* item that asked respondents if they believed that their community bonds together to solve problems	**Reliability:** The measures were not tested for reliability in the current study **Validity:** EFA was conducted and the six-factor solution explained 82.6% of the variance. All items had factor loadings greater than 0.4	Study used multilevel logistic regression models to examine the hypothesized relationships Results show: • Intergroup bridging ties associated with higher odds of four or more antenatal care visits (Odds Ratio (OR): 1.22; *p* < 0.05), and immunization of child (OR: 1.18; *p* < 0.05) • Intragroup bonding ties associated with lower odds of four or more antenatal visits (OR: 0.83; *p* < 0.05), immunization of child (OR: 0.86; *p* < 0.05) and higher odds of professional delivery care (OR: 1.19; *p* < 0.05) • Collective efficacy associated with higher odds of professional delivery care (OR: 1.12; *p* < 0.05), and lower odds of four or more antenatal care visits (OR: 0.90; *p* < 0.05)	GROUP DYNAMICS: • Group support • Group solidarity • Positive group dynamics • Social support and connection
9	Emery et al. [43]	To examine the associations of aspects related to partner control and gender norms with bystander intervention against intimate partner violence (IPV). Collective efficacy was included as a covariate in the analysis, defined as neighbourhood solidarity and neighbourhood informal social control	Cross-sectional Purpose: association analysis	Setting: China (Beijing) (Study also included a sample from a non-LMIC, South Korea; findings discussed here are for the sample from China only) Random probability proportional-to-size (PPS) cluster sample of 301 married/co-habiting women in China 94.68% were married Mean age: 42.82 years Mean education: 12.65 years	Collective efficacy was a covariate in this analysis, and included two sub-scales: neighbourhood **solidarity** and **neighbourhood informal social control** • **Neighbourhood solidarity:** includes *four* items which assess whether people in the neighbourhood care if the respondent has a problem, can be trusted, and are willing to help • **Neighbourhood informal social control:** includes *four* items which assess whether the woman can rely on their neighbours to do something if there was any violence or trouble in the neighbourhood	**Reliability**: • Neighbourhood solidarity: Cronbach’s alpha = 0.87 • Neighbourhood informal social control: Cronbach’s alpha = 0.86 **Validity:** No statistics for validity are provided in the current study	Study used multilevel regression models to test the hypothesized associations Results show: • Neighbourhood informal social control significantly associated with protective bystander behaviour against IPV in Beijing. Protective behaviour referred to intervening by trying to calm down the perpetrator of IPV by talking to them • No significant association observed for either of the two sub-scales of collective efficacy with punitive bystander behaviour, which referred to calling the police or threatening the perpetrator	GROUP DYNAMICS: • Group support • Group solidarity • Positive group dynamics • Social support and connection • Trust
10	Kumar et al. [7]	To examine whether group structure and process of women’s self-help groups (SHGs) are associated with the effectiveness of the SHG, with regards to its financial performance as well as relationships within the group Group structure and process are assessed in terms of group norms, participatory leadership, trust during financial transactions, group attendance, association with bank and federation, transparency, group cooperation and cohesion	Cross-sectional Purpose: association analysis	Setting: India Multi-stage sample of 2636 women from 210 functional SHGs Mean size (# of members) of the SHGs 12.5 Mean length of association with a bank 55.4 months. Only 1.2% of WSHG members from the General caste, 70.7% signature literate, and 58.7% involved in agricultural activities as their primary occupation	The key mediator in the analysis, **group structure and process**, refers to the underlying pattern of norms, roles, stable relationship patterns among members, and the interactive activities of group members among themselves and with the outside environment. It was assessed via the following variables • **Awareness about group norms**: includes *five* items that asked members about their awareness of meeting procedures, their roles, their leaders' roles, bookkeeping procedures, and about loans, savings, and fines • **Leadership**: includes *four* items that asked members about their closeness to leaders, their extent of leadership, and presence of leadership rotation within the group • **Trust in financial transactions**: includes *three* items that asked members about their trust on each other in depositing money and transactions with the bank, and their trust on leaders on financial decisions • **Co-operation**: includes *three* items that asked members if the group members support and help each other in different situations • **Group cohesiveness:** includes *four* items that asked members if the group prefers to work collectively or alone, and if given a chance they might join another group	**Reliability**: Cronbach’s alpha for the constructs related to collective efficacy not provided ICC for awareness about group norms, trust in financial transactions, and leadership ranged from 0.64 to 0.73 ICC for co-operation and group cohesiveness ranged from 0.68 to 0.77 **Validity:** Factor analysis generated a single factor solution that explained 82.86% of the total variance. All items had a factor loading of 0.90	Study used partial least squares structural equation modelling to assess the hypothesized relationships Results show: • Aspects related to collective efficacy (group structure and process) associated with effectiveness of women’s self-help groups (SHGs), with regards to its financial performance or profits made (t = 73.24; *p* < 0.001)	GROUP DYNAMICS: • Group support • Group solidarity • Trust COLLECTIVE ACTION • Leadership and voice
11	Lippman et al. [40]	To examine the association between community mobilization and incident HIV among adolescent girls and young women	Repeated cross-sectional Purpose: association analysis	Setting: South Africa Random sample of 2292 HIV‐negative adolescent girls and young women. (data collected in two rounds of surveys in 2012 and 2014) Mean age in 2012: 15.5 (SD 0.18). 26.6% have had sexual intercourse. At first round of data collection, 3.1% had engaged in transactional sex in past 12 months	Study uses a Community Mobilization Measure (CMM), which assesses efforts by a group/collective/community to take action towards achieving a shared goal. CMM is composed of seven domains: • **Shared concern:** includes *10* items that asks about shared concerns regarding HIV that can help community mobilize around this issue to improve access to resources, quality of care, and social inclusion • **Critical consciousness:** includes *11* items that captures any shared concerns in a community that connect people, and that people can mobilize to address • **Organizational structures and networks:** includes *14* items that examines the concrete elements in the community through which mobilization or organizing can occur, such as groups, platforms for information dissemination, or even buildings where people can convene, as well as those recognized by other structures to allow for voices to be elevated • **Leadership:** includes *10* items that assess both equity in opportunity to lead and have voice in mobilization efforts (i.e., inclusivity in leadership) and opportunity for the collective to take a leadership role in guiding change • **Collective action:** includes *six* items that asks about the efforts of collectivisation for social change • **Social cohesion:** includes *six* items that examines how much the members of the given group or collective connect with one another and align/identify with the group. This can be linked to shared trust or a sense of community or affiliation • **Social Control:** includes *eight* items that assesses how much community members are willing to intervene when community issues arise, for the public good. This is an informal means of social control	Measure was originally developed by Lippman et al., 2016^42^ for young adults in South Africa, via construct mapping for item development, inclusive of both formative research and expert inputs. Once the measure was developed cognitive interviews were conducted to ensure clarity and face validity, and then pilot tested it with 101 participants aged 18–35 in their target community. Following details are from the original and not the reviewed article **Reliability**: Raykov's ρ was used to assess reliability • Shared Concern about HIV in the community: Raykov's ρ = 0.85 • Critical Consciousness: Raykov's ρ = 0.93 • Leadership: Raykov's ρ = 0.92 • Organizations/Networks: Raykov's ρ = 0.81 • Collective Action: Raykov's ρ = 0.84 • Social Cohesion: Raykov's ρ = 0.81 • Social Control: Raykov's ρ = 0.89 **Validity:** Confirmatory factor analyses were conducted, based on findings from the EFA on pilot data. A seven-factor solution with good fit resulted from these analyses, which created the subscales for this measure * All scales except for the social control scale showed good inter-correlation, suggesting that six of seven scales represent community mobilization. Possibly, this is because the social control scale was originally developed for the United States and is not effectively capturing a meaningful construct in this context. (See Sampson et al., 2002 for original measures on social cohesion and social control.)	Study used logistic regression analysis to assess the hypothesized relationships Results show: • For every additional standard deviation of community mobilization at the village‐level, there was 12% lower HIV incidence (Risk Ratio (RR): 0.88, 95% CI: 0.79—0.98) after adjusting for individual, household and community characteristics. The specific scales of community mobilization associated with lower HIV incidence were critical consciousness (RR: 0.88; 95% CI: 0.79—0.97) and leadership (RR: 0.87; 95% CI: 0.79—0.95)	GROUP DYNAMICS: • Group support • Group solidarity • positive group dynamics • Social support and connection • Group dialogue • Identification with the group • Group decision-making/deliberation • Shared goals COLLECTIVE ACTION • Group organizing • Leadership and voice
12	Karlan et al. [41]	To evaluate a savings-led microfinance programme which aimed to improve financial services, microenterprise activity, income, female empowerment, consumption, and the ability to cope with shocks	Cluster RCT Purpose: evaluation	Setting: Ghana, Uganda, Malawi The evaluation included four surveys: household survey, adult survey, village survey, and market survey. For a sample of 13,502 households, adult women were interviewed (adult survey) to collect information on gender issues and community involvement	One of the outcomes of the analysis is the **community participation index**, which examines women’s involvement in community affairs, including raising an issue before any person of authority, and participation in community meetings and social groups	Not tested for reliability and validity	Study used independent ordinary least squares regression models, with a pooled model controlling for baseline values of the outcomes Results show: • The savings-led microfinance intervention had no significant impact on the construct related to collective efficacy—community participation of women. (intervention processes included enabling formation of savings group with regular meetings for decisions regarding contribution of savings)	GROUP DYNAMICS: • Group support • Group dialogue
13	Saggurti et al. [38]	To evaluate effects of a health behaviour change intervention with self-help groups (SHGs), aimed to increase women’s collective empowerment and improve MNCH practices among economically marginalized groups in India	Pre-post quasi-experimental Purpose: evaluation	Setting: India Multi-stage cluster sample of 545 SHG women who participated in intervention, in Bihar, India. (n = 374 intervention or WSHG members, n = 171 control) Mean (SD) age at baseline: 25 ± 5 years Education: Only 10% of the women had formal education	One of the outcomes for this evaluation study was **collectivisation,** assessed as including collective efficacy, agency, action and cohesion around maternal and child health • **Collective efficacy:** includes *four* items that assess whether the WSHG members believed in working together to bring positive changes around health • **Collective agency:** includes *three* items related to WSHG members assisting other members to seek/demand healthcare services or services from local administrative agencies • **Collective action:** includes *eight* items measuring the strategic and organized activities of SHGs to increase the members’ presence or enact its agenda for change • **Group cohesion** for health: includes *three* items related to provision or receipt of any maternal and child health services from an SHG member	Not tested for reliability and validity	Study used Difference—in—Difference (DID) to evaluate intervention effects Results show: • The behaviour change intervention with self-help groups (SHGs) increased collective efficacy levels among its members (DID: 17percentage points (pp), *p* < 0.001) • No significant impacts of the intervention observed for collective agency, collective action, and group cohesion	GROUP DYNAMICS: • Group support • Group solidarity • Positive group dynamics • Social support and connection • Group dialogue • Identification with the group • Group decision-making/deliberation • Shared goals COLLECTIVE ACTION • Group organizing
14	Gullo et al. [34]	To evaluate the effects of a community mobilization intervention on women's reports and experiences of health governance, defined as consisting of aspects such as trust in health workers, power sharing, mutual responsibility, collective efficacy, and the presence of a safe motherhood committee or community action group. Study also aimed to assess the relationship of these indicators of health governance with a) modern family planning, b) home visit from a community health worker, and c) satisfaction with health services	Two-armed cluster randomized controlled trial (RCT), Purpose: evaluation, secondary analysis	Setting: Malawi Cluster sample of 1300 women aged 15–49 who had given birth in the last 12 months (N = 651 in intervention [20 clusters], N = 649 in control [20 clusters]) 50% of the women under the age of 25 years. Less than two-thirds functionally literate. About 50% lived over one hour from the nearest health facility with basic emergency obstetric care available	One of the outcomes for this evaluation, **health governance,** was assessed via eight scales: • **Trust in health workers**: includes *six* items that asks about the degree to which respondents perceive health workers as caring, considerate, and attempting to provide the best care possible • **Power sharing:** includes *three* items that assess level of involvement, voice, and decision-making powers of community members and health workers • **Mutual responsibility:** includes *five* items that assess whether the respondents believe that women and health workers together have an impact on services between community and health workers • **Joint monitoring and transparency:** includes *six* items that assess whether community members and health workers identify and address concerns • **Equity and quality:** includes *six* items that assess the breadth of community participation, particularly from vulnerable groups, and perceptions of inclusivity • **Collective efficacy:** includes *five* items that assess women’s confidence in how well community members and health workers could work together • **Collective action:** includes *seven* items that assess the perceived improvement in maternal and child health care services because of collective action • **Perceived value of Governance Score Cards**: includes *five* items that assess the perceived improvement in maternal and child health care services because of the community intervention	**Reliability**: • Trust in health workers: Cronbach's alpha = 0.80 • Power sharing: Cronbach's alpha = 0.79 • Mutual responsibility: Cronbach's alpha = 0.65 • Joint monitoring and transparency: Cronbach's alpha = 0.93 • Equity and quality: Cronbach's alpha = 0.84 • Collective efficacy: Cronbach's alpha = 0.82 • Collective action: Cronbach's alpha = 0.93 • Perceived value: Cronbach's alpha = 0.92 **Validity:** Exploratory factor analysis (EFA) was conducted on the set of items which generated an 8-factor solution. EFA models were fit using Mplus with varimax rotation	Study used a local average treatment effect (LATE) analysis, and effects of the intervention were estimated based on compliance/attendance (rather than intent-to-treat) Results show: • At end line, intervention relative to control participants were more likely to report awareness of the Community Action Group or Safe Motherhood Committee (*p* = .03) • Among women in the intervention areas who were aware of the intervention, intervention participation was associated with trust in health workers (negative association, *p* = 0.49), mutual responsibility (negative association, *p* = 0.005), participation in negotiated spaces (*p* < .001), joint monitoring and transparency (*p* = 0.029), equity and quality (*p* < 0.001), collective action (*p* < 0.001), awareness of the Community Action Group or Safe Motherhood Committee (*p* < 0.002), and community help (*p* = 0.020) • Collective action was positively associated with home visits (*p* = .001) and modern family planning use (*p* = .05), but negatively associated with satisfaction with services (*p* = .009)	GROUP DYNAMICS: • Positive group dynamics • Group dialogue • Trust • Group decision-making/deliberation • Shared goals COLLECTIVE ACTION • Group organizing

Collective efficacy sub-constructs: Most of the studies included multiple measures, or measures capturing different sub-constructs of collective efficacy [32–40, 42–44]. Unique measures were seen across studies, with the exception of two articles that used data from the same study with FSWs in India [32, 33]. All 14 studies captured group dynamics; seven also included collective action.

We observed variance in the objectives and study design of the eligible research. Four studies focused on measures development and validation, with the remaining being association analyses or evaluation studies. Nonetheless, most (12 studies) provided some psychometric data to be included in our analyses. In the following section, we outline the key populations of focus, psychometrics, and key concepts of collective efficacy of focus for each study, by study design.

### Validation studies

As seen in the Rows 1–4 of Table 1, validation studies were conducted with women in Sri Lanka, Malawi and Iran, including pregnant women and new mothers, health workers, and the general female population [35, 36, 39, 42]. These studies captured sub-constructs of collective efficacy, including social capital, trust, and empowerment. All measures demonstrated good internal reliability and validity. Measures ranged in length from eight to 24 items, with the longer measures more comprehensively assessing group dynamics and connectivity. The measure from Sri Lanka with pregnant women assessed social capital, capturing elements of neighborhood networks, domestic and neighborhood cohesion, social contribution, and social participation [36]. A measure developed with young women in Iran assessed a single component of group dynamics- trust [42]. Only one of the four measures included a measure of collective action in the form of organizing, with a sample of new mothers in Malawi [35]. While this measure was a longer measure, with 24 items, it comprised of subscales to capture aspects of group support, group cohesion, perceptions of group capacity to have impact, and collective action. It included items measuring women's confidence in how well members can work together for a specific goal or impact, thus capturing the capacity to 'act together'.

### Association analyses

Seven studies focused on associations between collective efficacy and health and economic outcomes for women and girls (Table 1, Rows 5–11), with four studies capturing both group dynamics and collective action.

Three studies from Dominican Republic [31] and India [32, 33] focused on FSWs. The study in the Dominican Republic used an 11-item measure of a component of group dynamics—group cohesion (e.g., trust and solidarity with other FSWs); the measure demonstrated good internal reliability but was not tested for validity. This study showed a significant association between group cohesion and consistent condom use. The two studies from India used more comprehensive measures of collective efficacy, including items for both group dynamics, and collective action [32, 33]. These studies analyzed data from the same intervention, and hence had similar measures. A four-item measure of collective efficacy was used, that assessed whether the FSWs worked well together to achieve their shared goals. Studies also captured measures of collective agency (four items on standing up to someone in power to help another FSW), and collective action (six items on demanding entitlements via a group or collective of FSWs). The measures showed good internal reliability but were not tested for validity. However, the studies demonstrated significant associations between aspects of collective efficacy and consistent condom use, sexual self-efficacy, and willingness to engage in STI treatment. Another study from India found women’s social capital to have a significant positive relationship with maternal and child health outcomes [44]. Social capital was measured with 15 items, with six distinct factors that captured bridging and bonding ties, social networks and cohesion, and perceived community bonds for collective action. The measure was not tested for reliability but showed adequate validity, with the six factors explaining over 80% of the variance in factor analysis. One study with married or cohabiting women in China used an eight-item measure of collective efficacy with good reliability. It captured two factors related to group dynamics: neighborhood solidarity, and neighborhood informal social control [43]. The second factor, a measure of trust and perceived support from one’s neighborhood, was found to be significantly associated with women’s bystander behavior against intimate partner violence.

Another study used a 19-item measure to capture group dynamics, assessing group structure and processes of women’s SHGs (trust in the SHG’s processes, leadership, and group cohesiveness) in India [37]. The measure had good reliability and validity, and demonstrated a significant relationship between SHGs’ group structure and processes, and financial health of the SHGs.

A study from South Africa provided the most comprehensive assessment, with a measure for Community Mobilization, which included 55 items that assessed seven distinct aspects of collective efficacy across both group dynamics and collective action [40]. The results were quite robust, with higher community mobilization at the village level associated with lower HIV incidence. The subscales predictive of this outcome were critical consciousness (shared goals in the collective), and women’s leadership (an indicator of mobilization). The measure demonstrated very strong reliability and validity scores and was developed with both theory and rigorous science, described in the original measure development and validation study [45].

### Intervention evaluation studies

As seen in the Rows 12–14 of Table 1, three evaluation studies that included measures of collective efficacy examined the impact of SHGs and a community mobilization program. An evaluation study of an SHG program in India included measures for both our sub-constructs of collective efficacy- group dynamics and collective action [38]. This study used eight sub-scales to measure aspects of trust, positive group dynamics, group efficacy, and collective action or group organizing. Measure showed good reliability and validity, and results indicated a positive impact of the community mobilization program on key aspects related to collective efficacy. The study demonstrated a significant increase in women’s collective efficacy due to the program. The evaluation study of a health-related community mobilization intervention in Malawi presented more comprehensive measures and robust psychometric results [34]. This measure included questions on group engagement and dialogue, trust, shared goals, as well as on collective action and organizing.

## Discussion

The current systematic review suggests a growing literature on measurement of collective efficacy from LMICs, with 14 reviewed studies from diverse geographies and population groups. The studies were published on or after 2012, although our review period dates to 2009. We found all studies to include measures related to group dynamics, while seven studies also captured dimensions of collective action. All except two studies included unique measures of collective efficacy. This is the first review to identify and examine measures of collective efficacy for women from LMICs, a context where aspects related to community relationships and engagement have shown to be protective factors for women’s health [46, 47]. Overall, measures demonstrated good reliability and validity when tested [35, 36, 39, 42], and those testing for associations or effects found a positive relationship between collective efficacy and women’s health and economic outcomes. However, gaps remain, both in terms of availability of a standard measure, harmonization of the available measures, and expanding our knowledge regarding different elements of collective efficacy beyond aspects of group dynamics, covering group support and group solidarity.

Our review demonstrates heterogeneity in measures for collective efficacy. While the reviewed body of work indicates promising results, a lack of consistency in measurement instruments impedes the ability to harmonize findings. Only two of the 14 reviewed studies included similar measures. The current heterogeneity could be attributed to the studies measuring different elements or sub-constructs of the two building blocks of collective efficacy, as identified by us. Although, for the few studies measuring the same construct, such as social capital, measurement instruments varied [36, 39, 44]. This heterogeneity makes summarization of results difficult. It makes drawing of valid conclusions regarding the different elements of collective efficacy and its impact on specific health outcomes, challenging. Another aspect to consider with regards to results synthesis is geographical diversity of measures. Five of the 14 studies in our review were from India [32, 33, 37, 38, 44]. For countries where tested, validated, and robust measures already exist, future research should consider adaptation of these measures instead of investments in development of new measures. Alternately, countries with no prior validated measures could focus on testing or development of measures to contribute to a comprehensive understanding of collective efficacy across different populations.

Our review found a greater focus of existing literature on aspects of collective efficacy related to group dynamics. Cognitive aspects including perceived group support, group solidarity, and trust, were frequently included in the measurement instruments [31, 39, 42, 44]. Whereas, we observed less representation of studies on community mobilization and collective action. Other key elements of collective efficacy, including informal social control, and social regulation also need to be explored by future research [48]. Further, the reviewed studies examined collective efficacy as a static or cross-sectional construct. None of the studies assessed it as a dynamic construct, where the sense of efficacy could undergo revisions based on new information received from group members [49]. This idea, and its implications, have been studied to some extent in self-efficacy [50], but is yet to be accounted for, in collective efficacy literature.

Majority of the studies that aimed to test associations focused on health outcomes of women. We note only one study that looked at economic outcomes related to financial health of SHGs. Even with regards to health outcomes, the value of the current work is limited to reproductive, maternal, and child health outcomes. Other outcomes of importance which could be impacted by collective efficacy, are missing from the literature; these could include empowerment and individual agency, political leadership, and environment and sustainability [51, 52]. Our review also indicates gaps with regards to specific sub-populations, including adolescents and marginalized women groups. Thirteen of the 14 studies measured collective efficacy and its correlates among adult women. Adolescence is a formative life stage, and community related factors including collective efficacy during this period could have long-lasting health impacts for young individuals. Similarly, collective efficacy for marginalized groups might have different meanings and consequences, and is worth exploring.

While our study highlights important findings regarding measures of collective efficacy, it has few limitations. Our review included studies published in English; it is possible that inclusion of literature from other languages could increase the breadth of the review. Next, our review was limited to studies which included only women and girls in their sample, excluding work that focusses on measures of collective efficacy for a general population in LMICs. However, given the unique role of collectives on women’s health and well-being, as is evident from the research around SHGs and other collectives, a limited focus on measures that are developed specifically for women is necessary and critical. Third, our review focused on LMICs, where there might be a limitation in availability of research reports. Interventions on collective action, and community mobilization could be capturing these constructs in their monitoring or evaluation efforts, but not necessarily publishing findings as peer-reviewed literature. Finally, while the focus of this study was to provide insight into measures of collective efficacy, findings from this review also demonstrate the utility of collective efficacy with regards to its impacts on women's well-being. Future research should examine the associations between collective efficacy and key outcomes related to specific issues, such as health or economics, for further insight. Such focus was beyond the scope of the current study, but may prove important for more concrete guidance for future interventions.

## Conclusions

Collective efficacy is a key construct with regards to its implications for women’s well-being in low-resource settings. The current measurement science in this field shows promise, particularly for measures of one sub-construct of collective efficacy—group dynamics. Measures capturing collective action, or group organizing are still limited. While these measures can be adapted and applied in other settings, further research is needed to support harmonization and standardization of specific elements of collective efficacy, to focus on under-measured dimensions of collective efficacy, and to include broader segments of society with an intersectional lens.

## Supplementary Information

**Additional file 1:** Search strategy for the review.

## Data Availability

The datasets used and/or analyzed during the current study are available from the corresponding author on reasonable request.

## Abbreviations

LMIC: Low- and Middle-Income countries
STROBE: Strengthening the Reporting of Observational Studies in Epidemiology
EMERGE: Evidence based Measures of Empowerment for Research on Gender Equality
FSW: Female Sex Worker
SHG: Self-Help Group
