# Supplementary material for: Collective efficacy measures for women and girls in low- and middle-income countries: a systematic review
Source: BMC Womens Health. 2022 Apr 25;22:129. doi: 10.1186/s12905-022-01688-z (PMC9036723; doi:10.1186/s12905-022-01688-z)
Supplement: Supplementary file 1 — Additional file 1: Search strategy for the review. [file 12905_2022_1688_MOESM1_ESM.pdf]

Search strategy used for systematic review of collective efficacy measures for women and girls in low- and middle-income countries

community mobilization OR community participation OR community empowerment OR community capacity OR community connectedness OR collective efficacy OR collective action OR social cohesion OR social capital OR social capital OR community capital OR collectivization OR microfinance OR social efficacy OR community involvement

AND

(Surveys and Questionnaires [Mesh Term]) OR measure OR measures OR measuring OR scale OR scales OR index OR questionnaire OR questionnaires OR instrument OR survey*

AND

(Reproducibility of Results [Mesh Term]) OR (Validation Studies as Topic [Mesh Term]) OR (Validation Studies [Publication Type]) OR reliab* OR validat*

AND

(Developing Countries [Mesh Term]) OR low- and middle- income countries OR low-income countries OR middle-income countries[+]

[+] Also includes names of all low- and middle- income countries as search terms
